# Japanese Surgery and Medicine

**Published:** 1880-01

**Authors:** Edmund Andrews


### ARTICLE VIII.

### JAPANESE SURGERY AND MEDICINE.

*Messrs. Editors*:—I made an attempt, some time since to learn the state of surgery and medicine among the Japanese. The effort did not yield very brilliant results; yet the ideas of such a people are always of interest to thoughtful men, and are worthy of consideration in the study of human progress.

My information is derived partly from portions of an illustrated native Japanese work, translated for me by a lady, long resident in Japan, and partly from the kindness of Dr. H. Foulds, Physician of the Fukiji Hospital, in Tokio.

A favorite Japanese medicine for diseases of the alimentary canal is prepared by the tribe of Ainos, in the great island of Yezo, in the north part of the empire. These Ainos are a race of heavily bearded men, surrounded by the half beardless Asiatic races. Many ethnologists declare that in features and in physical structure they belong to the Caucasian race; but no one knows how they came to be flung off among the Mongolians, so many thousands of miles from the rest of the stock. However this may be, they were the aboriginal inhabitants of the whole of Japan, but about six hundred years before Christ they were invaded and driven north by the present dominant race of the empire, so that the Ainos are confined to the island of Yezo, and are only about fifty thousand in number. They pay a tribute of seals to the Japanese government, which they catch in the following curious manner: A decoy is made by taking a small float and erecting a sort of plume or bush upon it, which looks like a fox's tail. Three men go softly out in a canoe. One paddles the craft, another lets the float out with a string, which drifts away on the water, the third stands with a bow and arrows. The plan is to "toll" the animals. The seals, seeing the plume waving above the water, get their curiosity excited, much as the western antelopes do when "tolled" on land by an analogous device, and come swimming toward the plume. The man in the canoe draws the float gradually toward him, with the seals following it, until he gets them near enough to be shot by the archer.

The inland Ainos, however, are the ones who manufacture the popular medicine above referred to. They hunt female bears by first catching the cubs, and using them as decoys to catch the mother. They then take the heart, lungs, kidneys and liver, and, if I understand them rightly, make a sort of sausage meat by chopping them all up together, gall bladder included, and drying it in cakes. This is purchased as a medicine all over the empire, and is taken by simply cutting of slices and eating them. It is said by the patients to be "very bitter, like the gall of a chicken."

The aboriginal Japanese surgery was very rude, and had no anatomical knowledge as a basis. They used the moxa; and our word is simply a modification of a China-Japanese word for the same thing, viz., "Mō-hŭsa."

They bleed, and use a style of lancet supposed to be derived from the old Dutch traders two hundred years ago. Syphilis was known and described three centuries ago, but it is not clear what the most ancient treatment was. It is certain, however, that foreign surgeons found them not long since using enormous doses of mercury, so that the deleterious effects of the drug were very common.

Their peculiarly tough and porous paper is very valuable in surgery. Dr. Foulds uses it for bandages, wrappings, compresses and absorbents, as well as for holding antiseptic applications, and also for sponges and towels.

The doctor thinks the utter ignorance of anatomy shown in Japanese medical works is due to the horror of taking animal life inculcated by the Buddhist religion, by which investigators were prevented from even dissecting inferior animals to obtain light on human structure.

On the whole, the Japenese do not seem likely to furnish any valuable new ideas in medicine or surgery, but the flora of their country is extremely rich and varied, and it is probable that a thorough and skilful examination of their native plants would furnish useful additions to our materia medica.

EDMUND ANDREWS, M.D.

